# Sodium-glucose cotransporter 2 inhibitors: a practical guide for the Dutch cardiologist based on real-world experience

**DOI:** 10.1007/s12471-021-01580-9

**Published:** 2021-06-16

**Authors:** K. Zwart, S. Velthuis, Y. V. Polyukhovych, A. Mosterd, L. Smidt, E. H. Serné, D. H. van Raalte, P. J. M. Elders, M. L. Handoko, P. C. Oldenburg-Ligtenberg

**Affiliations:** 1 Department of Internal Medicine/Endocrinology, Meander Medical Centre, Amersfoort, The Netherlands; 2 Department of Cardiology, Meander Medical Centre, Amersfoort, The Netherlands; 3 Department of Cardiology, Amsterdam University Medical Centers, location VU University Medical Center, Amsterdam, The Netherlands; 4 Department of Internal Medicine/Endocrinology, Amsterdam University Medical Centers, location VU University Medical Center, Amsterdam, The Netherlands; 5 Department of General Practice, Amsterdam University Medical Centers, location VU University Medical Center, Amsterdam, The Netherlands

**Keywords:** SGLT2 inhibitor, Cardiologist, Guide, Flowchart, Real-world evidence

## Abstract

Sodium-glucose cotransporter 2 (SGLT2) inhibitors include a relatively new class of glucose-lowering drugs that reduce plasma glucose concentrations by inhibiting proximal tubular reabsorption of glucose in the kidney, while increasing its excretion in urine. Recent large randomised controlled trials have demonstrated that many of these agents reduce the occurrence of major adverse cardiovascular events, hospitalisation for heart failure, cardiovascular death and/or chronic kidney disease progression in patients with and without type 2 diabetes mellitus (DM2). Given their unique insulin-independent mode of action and favourable efficacy and adverse-event profile, SGLT2 inhibitors are promising and they offer an interesting therapeutic approach for the cardiologist to incorporate into routine practice. However, despite accumulating data supporting this class of therapy, cardiologists infrequently prescribe SGLT2 inhibitors, potentially due to a lack of familiarity with their use and the reticence to change DM medication. Here, we provide an up-to-date practical guide highlighting important elements of treatment initiation based on real-world evidence and expert opinion. We describe how to change DM medication, including insulin dosing when appropriate, and how to anticipate any adverse events based on real-world experience in patients with DM2 in the Meander Medical Centre in Amersfoort, the Netherlands. This includes a simple algorithm showing how to initiate SGLT2 inhibitor treatment safely, while considering the consequence of the glucosuric effects of these inhibitors for the individual patient.

**Supplementary Information:**

The online version of this article (10.1007/s12471-021-01580-9) contains supplementary material, which is available to authorized users.

## What’s new?

- Sodium-glucose cotransporter 2 (SGLT2) inhibitors include a relatively new class of glucose-lowering drugs.
- Recent large randomised controlled trials have demonstrated that many of these agents reduce the occurrence of major adverse cardiovascular events, hospitalisation for heart failure, cardiovascular death, and/or chronic kidney disease progression, *regardless* of the presence or absence of diabetes mellitus.
- We provide an up-to-date practical guide for the cardiologist, highlighting important elements for treatment initiation based on real-world evidence and expert opinion, including a simple algorithm that shows how to initiate SGLT2 inhibitor treatment safely.

## Introduction

Sodium-glucose cotransporter 2 (SGLT2) inhibitors have emerged as an important new oral glucose-lowering class of drugs for the management of hyperglycaemia in patients with type 2 diabetes mellitus (DM2). Importantly, recent large randomised controlled trials have demonstrated that many of these agents reduce the occurrence of major adverse cardiovascular events, hospitalisation for heart failure (HF), cardiovascular death and/or progression of chronic kidney disease (CKD), regardless of the presence or absence of DM2 [1–9]. These findings, combined with the beneficial overall safety profile, make SGLT2 inhibitors an interesting therapeutic approach for the cardiologist. However, despite accumulating data supporting this new class of therapy, cardiologists infrequently prescribe SGLT2 inhibitors, potentially due to a lack of familiarity with their use and concern about prescribing them in combination with other glucose-lowering medications, such as insulin therapy. A relatively simple flow chart can be designed to give most cardiologists more confidence and the assurance they are not going to do harm.

In this article, we provide an up-to-date practical guide for the Dutch cardiologist, highlighting important elements for treatment initiation, dosing, anticipated adverse events and barriers, based on expert opinion and real-world experience in patients with DM2 in the Meander Medical Centre in Amersfoort, the Netherlands. It includes a simple algorithm showing how to initiate SGLT2 inhibitor treatment safely, while considering the consequence of the glucosuric effects of these inhibitors for the individual patient.

## Mechanism of action

The SGLT2 receptor is a sodium-glucose cotransporter located in the proximal tubule of the nephron that is responsible for approximately 90% of urinary glucose reabsorption. Inhibition of this receptor results in lower blood glucose levels through induction of glucosuria [10]. This effect is more pronounced in the setting of hyperglycaemia, when significant amounts of glucose are filtered into the urine. Glucosuria diminishes significantly as blood glucose levels normalise. Furthermore, as the estimated glomerular filtration rate (eGFR) decreases, the effects of inhibiting the SGLT2 receptor on blood glucose levels are smaller. The risk of hypoglycaemia in patients on an SGLT2 inhibitor is therefore extremely low, unless this agent is taken concomitantly with insulin or sulfonylureas.

**Fig. 1:**
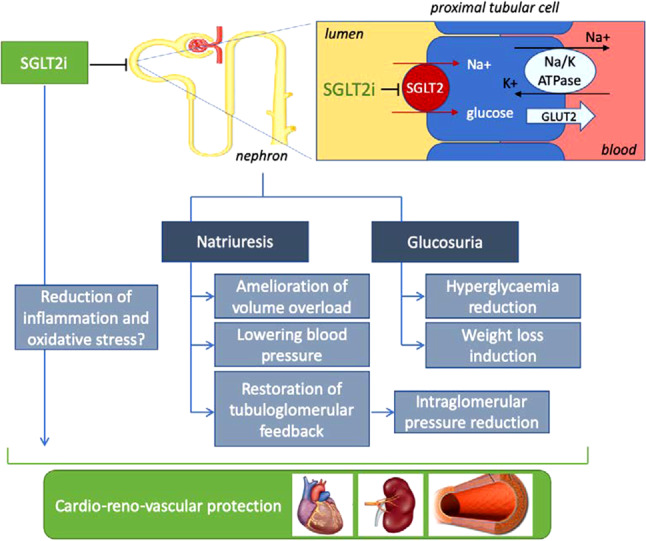
Mechanism of effects of sodium-glucose cotransporter 2 inhibitor (*SGLT2i*). *GLUT2* glucose transporter 2

Beyond their effect on blood glucose levels, SGLT2 inhibitors also cause diuretic and possibly natriuretic effects, promote weight loss and lower systolic blood pressure (Fig. 1; [10]). Although the mechanism of effects of SGLT2 inhibitors have not been fully elucidated, it is largely independent of lowering HbA_1c_ levels, and a number of putative mechanisms have been proposed [10].

## Major adverse cardiovascular events

Large randomised controlled trials in patients with DM2 have demonstrated that many of these agents reduce major adverse cardiovascular event endpoints in patients with established atherosclerotic cardiovascular disease and/or CKD and reduce the risk of HF hospitalisations (see Table S1 in the Electronic Supplementary Material) [1–9]. These benefits may be similar for agents within this class, although there are differences that are likely to reflect the patient populations enrolled in the trials [1–8].

SGLT2 inhibitors have moderate effects (risk reduction ~14%) on atherosclerotic major adverse cardiovascular events that seem confined to patients with established atherosclerotic cardiovascular disease or CKD (eGFR < 60 mL/min per 1.73 m^2^) [11].

## Heart-failure events

At first, the benefit of reducing the number of HF hospitalisations in SGLT2 inhibitor trials [1–6] primarily reflected prevention of symptomatic HF in DM2 patients at high risk, as approximately 90% did not have HF at baseline (and those who did, were not well characterised). The effects of SGLT2 inhibitors on HF hospitalisation appeared to be remarkably consistent across the class (approximately a 30% reduction in hospitalisation for HF; see Table S1 in the Electronic Supplementary Material).

More recently, the benefits of an SGLT2 inhibitor in treating established HF were demonstrated in the DAPA-HF (Study to Evaluate the Effect of Dapagliflozin on the Incidence of Worsening HF or Cardiovascular Death in Patients with Chronic HF) and the EMPEROR-Reduced (Cardiovascular and Renal Outcomes with Empagliflozin in Heart Failure) trial [7, 8]. Both studies enrolled patients with HF failure with reduced ejection fraction (HFrEF) with and without DM who were receiving appropriate background treatments for HF. A recent meta-analysis of these two trials reported that in patients with a broad spectrum of HFrEF severity, SGLT2 inhibition with empagliflozin or dapagliflozin, on top of guideline-directed medical therapy, reduced all-cause and cardiovascular death, HF hospitalisations and serious adverse renal outcomes, without heterogeneity between the two trials [12].

The pooled treatment effects showed consistent benefits for subgroups based on age, sex, presence of DM, baseline eGFR or background treatment with an angiotensin receptor-neprilysin inhibitor, but suggested treatment-by-subgroup interactions for subgroups based on the New York Heart Association (NYHA) functional class and possibly race. The pooled hazard ratio for patients in NYHA class II (0.67, 95% confidence interval (CI) 0.59–0.76) differed from that for patients in class III–IV (0.87, 0.75–1.01). Beneficial effects on symptoms, functional status and quality of life in patients with HFrEF were also reported. Additional trials with various agents in patients with HF with preserved ejection fraction are ongoing.

## Renal events

In addition, consistent reductions in the secondary outcome of risk of kidney disease progression were seen with all agents in the cardiovascular outcome trials, although the number of ‘hard’ renal events (e.g. progression to end-stage renal disease and renal death) was small. The CREDENCE (Evaluation of the Effects of Canagliflozin on Renal and Cardiovascular Outcomes in Participants with Diabetic Nephropathy) trial [5] and DAPA-CKD (Dapagliflozin in Patients with Chronic Kidney Disease) trial [6]—both dedicated renal outcome trials involving the SGLT2 inhibitor class—reported that both canagliflozin and dapagliflozin significantly reduced the composite of a sustained eGFR decline ≥ 50%, end-stage kidney disease or death from renal or cardiovascular causes. The DAPA-CKD trial demonstrated that these beneficial renal effects were similar in participants with and without DM2.

Mechanisms to explain these observations may include activation of tubuloglomerular feedback, reduction in glomerular hypertension, containment of hyperfiltration injury, reduction of kidney hypoxia and possible effects on sodium-hydrogen exchange [10, 13].

## Other adverse events

An increased risk of genital mycotic infections (mostly candida vaginitis in women, balanitis in men) has been seen with all SGLT2 inhibitors [11]. These infections are usually not harmful and tend to resolve after a brief course of antifungal therapy, although careful education and monitoring are imperative in patients considered to be at high risk of infectious complications. Rare reports of necrotising fasciitis of the perineum (Fournier’s fasciitis) led the Food and Drug Administration (FDA) to request the addition of a warning to the SGLT2 inhibitor prescribing instructions; whether these very rare but serious infections are causally related to SGLT2 inhibitor therapy remains unclear [14].

An increased risk of amputations and fractures was observed in one trial during treatment with canagliflozin; however, this phenomenon was not seen in other trials or in the other canagliflozin trial (CREDENCE). The clinical importance of any possible increase in amputation risk is also unknown, but caution is suggested in those with a history of peripheral artery disease and/or lower extremity diabetic ulcers. A recent meta-analysis indicated that SGLT2 inhibitors do not increase the risk of bone fracture in patients with DM2 compared with placebo [15]. There was a small risk of diabetic ketoacidosis (DKA), which may present itself in the absence of significant hyperglycaemia—often called ‘euglycaemic DKA’ [16]. Patients with DM2 who have progressed to an insulin-deficient state may develop euglycaemic DKA as a result of SGLT2 inhibitor therapy. The risk of developing DKA was almost two times higher in patients with DM2 taking SGLT2 inhibitors than in those taking placebo (2.20, 95% CI 1.25–3.87), but the event rates were low (< 1 per 1000 patient-years) [11].

A recent meta-analysis showed no increased risk of DKA in patients with DM2 taking an SGLT2 inhibitor compared with placebo [17]. In general, DKA predominantly renders a significant problem in DM1 patients who are treated with SGLT2 inhibition. However, it should be emphasised that DKA can occur in patients with DM2 and awareness of this complication is important. Lastly, the osmotic diuretic effect of SGLT2 inhibitors could lead to volume depletion and electrolyte imbalances, especially in patients with malnutrition and/or low intake.

## National and international guidelines

The 2019 Clinical Practice Guidelines by the European Society of Cardiology (ESC) on DM recommend either SGLT2 inhibitors or glucagon-like peptide‑1 receptor agonists (GLP‑1 RAs) in patients with DM2 and established atherosclerotic cardiovascular disease or in case of very high/high cardiovascular risk (e.g. ≥ 3 major risk factors or DM duration ≥ 10 years), to reduce cardiovascular events [18]. In addition, SGLT2 inhibitors are recommended to lower risk of HF hospitalisation if eGFR > 30 mL/min per 1.73 m^2^.

The 2019 version of ‘Management of Hyperglycemia in Type 2 Diabetes’, a consensus report by the American Diabetes Association and the European Association for the Study of Diabetes, offers more direction on when to prescribe SGLT2 inhibitors or GLP‑1 RAs in patients with DM2 at high or very high cardiovascular risk [19]. The level of evidence for benefit of SGLT2 inhibitors is greatest for patients with or without established atherosclerotic cardiovascular disease but with HFrEF (left ventricular ejection fraction (LVEF) < 40%) or CKD (eGFR 30–60 mL/min per 1.73 m^2^ or urine albumin:creatinine ratio (UACR) > 30 mg/g, particularly when UACR ≥ 300 mg/g).

Most guidelines do not address patients without DM2. The Canadian Cardiovascular Society/Canadian Heart Failure Society’s Heart Failure Guidelines are among the first to recommend that SGLT2 inhibitors be prescribed in patients with HFrEF (LVEF < 40%) and without concomitant DM, to improve symptoms and quality of life and to reduce the risk of hospitalisation and cardiovascular mortality [20]. The in 2021 updated version of the expert consensus decision pathway by the American College of Cardiology also recommends SGLT2 inhibitor therapy in patients with HFrEF with or without DM2 [21]. An update of the ESC guideline is expected next year. Cardiologists should consider adding an SGLT2 inhibitor in patients with HFrEF and without DM2; however, our real-world evidence is based on patients with DM2.

Dutch guidelines on DM, CKD and HF are currently being updated. The Dutch Diabetic Nephropathy guideline recommends SGLT2 inhibitor therapy in two patient groups: (1) eGFR 30–60 mL/min per 1.73 m^2^ and UACR > 3 mg/mmol, and (2) eGFR ≥ 60 mL/min per 1.73 m^2^ and UACR > 30 mg/mmol [22].

## Algorithm for SGLT2 inhibitor treatment initiation

SGLT2 inhibitor therapy can be considered for patients with the following characteristics:

- DM2 and a very high cardiovascular risk:
  1. Established atherosclerotic cardiovascular disease: prior myocardial infarction, ischaemic stroke, unstable angina with electrocardiographic changes, myocardial ischaemia on imaging or stress test, or revascularisation of coronary, carotid, or peripheral arteries.
  2. Chronic kidney disease^1^ (eGFR 30–60 mL/min per 1.73 m^2^ and UACR > 3 mg/mmol or eGFR ≥ 60 mL/min per 1.73 m^2^ and UACR > 30 mg/mmol).
- HFrEF (LVEF < 40%)

Although the FDA-approved cardiovascular indications are different, SGLT2 inhibitors appear to have broadly similar cardiovascular and renal benefits (see Table S1 in the Electronic Supplementary Material). A summary of the doses, indications, contraindications and adverse effects of SGLT2 inhibitors is shown in Table S2 (see Electronic Supplementary Material).

**Fig. 2:**
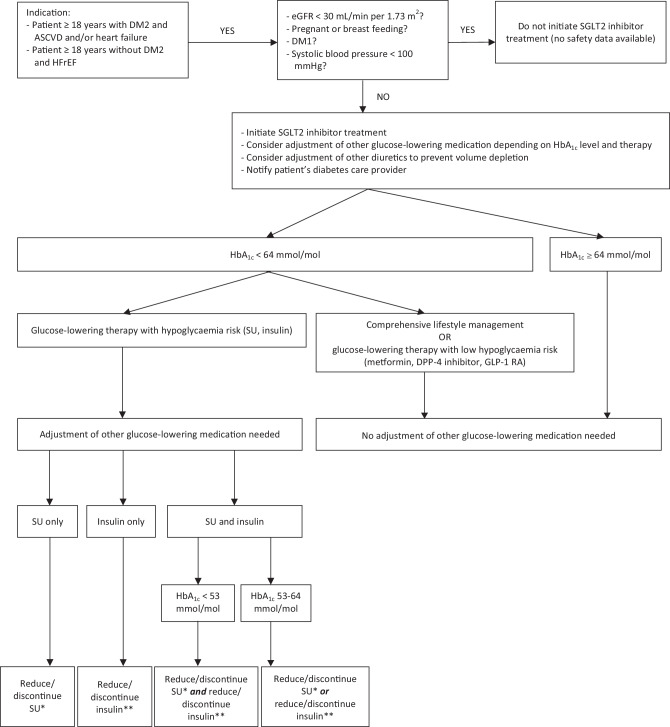
Flow chart of initiation of sodium-glucose cotransporter 2 (*SGLT2*) inhibitor treatment. *DM2* type 2 diabetes mellitus, *ASCVD* atherosclerotic cardiovascular disease, *eGFR* estimated glomerular filtration rate, *DM1* type 1 diabetes mellitus, *SU* sulfonylurea, *DPP‑4* dipeptidyl peptidase‑4, *GLP‑1 RA* glucagon-like peptide‑1 receptor agonist. *Discontinue sulfonylurea if patient is on gliclazide ≤ 80 mg once daily/glimepiride ≤ 2 mg once daily/tolbutamide ≤ 500 mg twice daily. Otherwise, reduce sulfonylurea dose by 50%. **If patient is on insulin ≤ 12 IU/day, discontinue insulin. If patient is on insulin > 12 IU, reduce every insulin dose by 20%

Mostly, SGLT2 inhibitor treatment can be initiated safely by a cardiologist (Fig. 2). However, the following recommendations should be considered:

- The patient’s diabetes care provider (general practitioner, internist, nephrologist) should always be notified with a letter stating the clinical importance of SGLT2 inhibitors, any changes in diabetes medication, the possibility of adverse events (with specific attention to DKA) and the method of follow-up.
- SGLT2 inhibitors should not be prescribed in patients with DM1. If the type of DM has not be established, we advise to consult an internist, who may suggest diabetes autoantibody tests.
- Prescribers should be aware of precipitating factors of ketoacidosis (e.g. insulin cessation, prednisone administration, dehydration, hyperglycaemia, low carbohydrate intake/low food intake, excessive alcohol use). They should educate patients about the signs or symptoms of ketoacidosis (nausea, vomiting, abdominal pain, weakness) and instruct patients to discontinue SGLT2 inhibitors and seek immediate medical attention in case of such complaints. If there is (a low) clinical suspicion of DKA, we recommend consulting an internist for further advice.
- If HbA_1c_ ≥ 64 mmol/mol or the patient is treated with comprehensive lifestyle management only or in combination with glucose-lowering therapy with a low risk of hypoglycaemia (metformin, dipeptidyl peptidase‑4 inhibitors, GLP‑1 RAs), SGLT2 inhibitor treatment can be initiated safely without modifications.
- If HbA_1c_ < 64 mmol/mol and the patient is treated with glucose-lowering therapy with an increased risk of hypoglycaemia (i.e. sulfonylureas, insulin), SGLT2 inhibitor can only be initiated after adjustment of these glucose-lowering agents:
  - If patient is on a sulfonylurea drug but not on insulin, discontinue this therapy if patient is on:
    a) gliclazide ≤ 80 mg once daily
    b) glimepiride ≤ 2 mg once daily
    c) tolbutamide ≤ 500 mg twice daily
    Otherwise, reduce sulfonylurea dose by 50%.
  - If patient is on insulin but not on a sulfonylurea drug, reduce every insulin dose by 20%. Discontinue insulin when insulin dose ≤ 12 IU.
  - If patient is on a combination of sulfonylurea and insulin, and:
    a) HbA_1c_ ≤ 53 mmol/mol: reduce or discontinue sulfonylurea drug as stated above *and* discontinue insulin when insulin dose ≤ 12 IU. If insulin dose > 12 IU, reduce insulin dose by 20%.
    b) HbA_1c_ 54–64 mmol/mol: reduce or discontinue sulfonylurea drug as stated above *or* adjust insulin as stated above.
  - If there is uncertainty about changing the insulin dose, we recommend consulting an internist for further advice.
- SGLT2 inhibitors increase the risk of genital mycotic infections, polyuria, and potential volume depletion in the context of hyperglycaemia.
- Patients should be educated about the risk of genital mycotic infections and the importance of genital hygiene (e.g. keeping the genital region dry, especially after urinating). In most cases, genital infections will resolve after standard antifungal therapy without discontinuation of the diabetes medication regimen. If necessary, the therapy can be temporarily interrupted.
- It is prudent to educate patients about the signs and symptoms of dehydration (light-headedness, orthostatic hypotension, weakness), which may be more of a concern outside the clinical trial setting, especially in frail patients. Clinical judgement should be used when initiating SGLT2 inhibitor treatment in patients who will be undergoing renin-aldosterone-angiotensin system inhibition therapy (or in whom this dose is up-titrated) if the patient’s renal function is impaired. SGLT2 inhibitors should be discontinued in case of acute kidney injury as with other medications such as sulfonylureas, angiotensin converting enzyme inhibitors, diuretics, metformin, angiotensin receptor antagonists and non-steroidal anti-inflammatory drugs.
- Initiation of SGLT2 inhibitor treatment may lead to a transient acute decline of eGFR at week 4, followed by a period of stable kidney function during long-term follow-up, as was shown in the EMPA-REG OUTCOME trial; eGFR gradually declined with placebo [1].
- Follow-up by the diabetes care provider is essential after two weeks if the patient is on insulin and after six weeks if the patient is not taking insulin.
- The clinical importance of any possible increase in amputation risk remains unclear. It is recommended to examine the feet of all patients on foot wounds and diabetic ulcers with even more awareness for this issue in patients with a history of peripheral artery disease and lower extremity diabetic ulcers. If there is clinical uncertainty about the extent of peripheral artery disease, consult a vascular surgeon. Do not start or discontinue SGLT2 inhibitors if foot wounds and/or ulcers are present.

## Real world evidence of efficacy of proposed algorithm

Experience with the proposed algorithm as shown in Fig. 2 has been obtained in an observational prospective cohort study in the Meander Medical Centre in Amersfoort, with the small difference that an eGFR cut-off value of < 45 mL/min per 1.73 m^2^ was used previously instead of < 30 mL/min per 1.73 m^2^. We changed the eGFR cut-off value after completion of this study and after publication of the DAPA-CKD and EMPEROR-Reduced trial results, which showed beneficial effects of SGLT2 inhibitor treatment on renal function [6, 8].

From 15 November 2019 until 1 November 2020, 84 patients were included and observed for follow-up. The median follow-up period was 143 days. Baseline characteristics are summarised in Tab. 1. Patients on insulin therapy were seen by the diabetes nurse specialist at the outpatient clinic 1 to 2 weeks after treatment initiation. All patients who started taking an SGLT2 inhibitor, were seen by the internist 6 weeks after initiation.

**Table 1:** Baseline characteristics of the Meander Medical Center cohort

Variable	Patients (*N* = 84)
*Patient characteristics*	
Male	62 (74)
Age, in years	70
BMI, in kg/m^2^	29
*Location of SGLT2 inhibitor treatment initiation*	
Hospital admission	35 (42)
Outpatient appointment	49 (58)
*Type of SGLT2 inhibitor*	
Empagliflozin	67 (80)
Dapagliflozin	17 (20)
*Cardiovascular disease*^a^	84 (100)
Myocardial infarction	53 (63)
CABG	27 (33)
Coronary stenosis > 70%	73 (87)
Peripheral arterial disease	12 (14)
Heart failure, type	20 (23)
– Ischaemic	13 (16)
– Non-ischaemic	5 (6)
– Unknown aetiology	2 (2)
*Diabetes mellitus diagnosis*	84 (100)
De novo	3 (4)
Diabetic treatment (previously) prescribed by GP	72 (86)
*Diabetic drugs*	
Metformin	73 (87)
Sulfonylureas	35 (42)
DPP‑4 inhibitors	5 (6)
GLP‑1 RAs	4 (5)
Short-acting insulin	16 (19)
Long-acting insulin	23 (27)
*Cardiovascular drugs*	
Beta blockers	63 (75)
Diuretics	41 (49)
Calcium channel blockers	26 (31)
ACE inhibitors	34 (41)
Angiotensin II receptor blockers	14 (17)
Alpha blockers	3 (4)
ARNI	7 (8)
*Anticoagulants*	
Platelet aggregation inhibitors	61 (73)
Direct oral anticoagulants	22 (26)
Vitamin K antagonists	10 (12)
*Cholesterol-lowering drugs*	
HMG-CoA-reductase inhibitors	73 (87)
Cholesterol absorption inhibitors	11 (13)
PCSK9 inhibitors	2 (2)
None	7 (8)

Data are *n* (%) or mean

*BMI* body mass index, *SGLT2* sodium-glucose cotransporter 2, *CABG* coronary artery bypass grafting, *GP* general practitioner, *DPP‑4* dipeptidyl peptidase‑4, *GLP‑1 RA* glucagon-like peptide‑1 receptor agonist, *ACE* angiotensin-converting-enzyme, *ARNI* angiotensin receptor-neprilysin inhibitor, *HMG-CoA* 3-hydroxy-3-methyl-glutaryl coenzyme A, *PCKS9* proprotein convertase subtilisin/kexin type 9

^a^ Patient’s medical history may include multiple cardiovascular comorbidities

Based on the algorithm, the cardiologist changed glucose-lowering medication in 19 of the 84 patients (23%) (16 times sulfonylurea, 3 times long-acting insulin). In one of these patients, the internist further reduced the long-acting insulin dose based on the HbA_1c_ level. The long-acting insulin dose was reduced in other 11 cases and discontinued in 3 cases by the internist, again based on HbA_1c_ level targets and not due to hypoglycaemic events. Mean HbA_1c_ level reduced from 62 to 57 mmol/mol. Mean eGFR decreased from 73 to 69 mL/min per 1.73 m^2^, which is expected in the initial phase of SGLT2 inhibitor treatment.

In total, 20 adverse events (24%) were reported, including 7 urogenital infections (Tab. 2). Adverse events led to permanent discontinuation of an SGLT2 inhibitor in 8 patients. One patient underwent an amputation of the third digit of the right foot 2.5 weeks after initiation of SGLT2 inhibitor treatment. Neither the presence of foot wounds or diabetic ulcers nor patient’s symptoms were mentioned in the patient’s file upon treatment initiation. In retrospect, this patient did have newly diagnosed peripheral artery disease after he started taking the SGLT2 inhibitor. This emphasises the importance of actively asking patients about the presence of any foot wounds or complaints before initiating SGLT2 inhibitor treatment. Another adverse advent was an HF exacerbation after diuretic dose reduction subsequent to SGLT2 inhibitor treatment initiation. Caution is warranted when the diuretic dose is reduced upon initiating SGLT2 inhibitor treatment.

**Table 2:** Adverse events in the Meander Medical Center cohort

Adverse events	*n* (%)
*Total*	20 (24)
Urogenital infection	7 (8)
– Genital	6 (7)
– Urinary tract	1 (1)
Pruritus	4 (5)
Polyuria and polydipsia	3 (4)
General discomfort	1 (1)
Exacerbation of heart failure	1 (1)
Severe obstipation	1 (1)
Foot amputation	1 (1)
Mild hypoglycaemia^a^	1 (1)
*After permanent discontinuation of SGLT2 inhibitor treatment*	11 (13)
Urogenital infection	5 (6)
Foot amputation	1 (1)
Exacerbation of heart failure	1 (1)
General discomfort	1 (1)
Patient’s wish (not related to adverse events)	3 (4)

Data are number (*n*) of adverse events; % is percentage in total patient cohort (*N* = 84)

*SGLT2* sodium-glucose cotransporter 2

^a^ Serum glucose level temporarily < 3.9 mmol/L, no help from others needed

A 63-year-old, obese male with a medical history of atrial fibrillation and coronary artery bypass grafting died in his sleep for unknown reasons and did not have any prodromal symptoms 6 months after SGLT2 inhibitor treatment initiation. Sudden death is not a known adverse effect of SGLT2 inhibitors [1–9].

Six patients were lost to follow-up early on, because they wished to return to their general practitioner for diabetic care.

Statistical analysis of our observational non-randomised data was not possible and conclusions based on statistically significant differences could therefore not be drawn. However, our results do show preliminary safety in this population, and our study can serve as a valuable experience for cardiologists wanting to prescribe SGLT2 inhibitors in patients with DM2.

## Conclusion

This article provided a practical guide for cardiologists on initiating and monitoring of SGLT2 inhibitor treatment. Although we intended to facilitate clinical decision-making, the information provided in this article should complement, rather than supersede, good clinical judgement. Study data from our hospital suggest that using the algorithm presented was safe and gave cardiologists enough confidence and the assurance they were not going to do harm. It is important to strive for good multidisciplinary cooperation, not only between the departments of Cardiology and Internal medicine, but also with general practitioners and pharmacists.

We anticipate that our algorithm changes as new evidence emerges, but the goal is still to safely improve cardiovascular outcomes in patients with DM2 at very high risk of atherosclerotic disease.

## Supplementary Information

**Table S1. **SGLT2 inhibitors: Summary of the published SGLT2 inhibitor cardiovascular and renal outcomes trials

**Table S2.** Summary of currently available SGLT2 inhibitors in the Netherlands on indications, contra-indications, dosing, and most important side-effects

## Footnotes

1 Summary of product characteristics states that SGLT2 inhibitor treatment should not be initiated in patients with eGFR < 60 mL/min per 1.73 m^2^ because the glycaemic efficacy depends on renal function. This label will be changed as the beneficial cardiovascular effects do not depend on renal function.
