# Supplementary material for: Sodium-glucose cotransporter 2 inhibitors: a practical guide for the Dutch cardiologist based on real-world experience
Source: Neth Heart J. 2021 Jun 16;29(10):490–9. doi: 10.1007/s12471-021-01580-9 (PMC8455761; doi:10.1007/s12471-021-01580-9)
Supplement: Supplementary file 1 — Table S1. SGLT2 inhibitors: Summary of the published SGLT2 inhibitor cardiovascular and renal outcomes trials [file 12471_2021_1580_MOESM1_ESM.docx]

**Table S1.** SGLT2 inhibitors: Summary of the published SGLT2 inhibitor cardiovascular and renal outcomes trials

|  | **EMPA-REG OUTCOME**  **(2015)** | **CANVAS/CANVAS-R**  **(2017)** | **DECLARE-TIMI 58**  **(2018)** | **VERTIS**  **(2020)** | **CREDENCE**  **(2019)** | **DAPA-CKD**  **(2020)** | **DAPA-HF**  **(2019)** | **EMPEROR-**  **REDUCED**  **(2020)** |
| --- | --- | --- | --- | --- | --- | --- | --- | --- |
| **Patients, n** | 7,020 | 10,142 | 17,160 | 8,246 | 4,401 | 4,304 | 4,744 | 3,730 |
| **Age (years)** | 63.1 | 63.3 | 63.9 | 64.4 | 63.0 | 61.8 | 66.4 | 67.2 |
| **Drug** | Empagliflozin | Canagliflozin | Dapagliflozin | Ertugliflozin | Canagliflozin | Dapagliflozin | Dapagliflozin | Empagliflozin |
| **Dose** | 10 or 25 mg PO daily | 100 or 300 mg  PO daily | 10 mg  PO daily | 5 mg or 15 mg PO daily | 100 mg  PO daily | 10 mg  PO daily | 10 mg  PO daily | 10 mg  PO daily |
| **follow-up (years)** | 3.1 | 2.4 | 4.2 | 3.5 | 2.6 | 2.4 | 1.5 | 1.3 |
| **Diabetes (%)** | 100 | 100 | 100 | 100 | 100 | 67.6 | 41.8 | 49.8 |
| **ASCVD (%)** | 99 | 72 | 41 | 100 | 50 | 37.8 | Not reported | 52.8 |
| **Baseline HF (%)** | 10 | 14 | 10 | 24 | 15 | 10.9 | 100 | 100 |
| **NYHA**  **II**  **III**  **IV** | Not reported | Not reported | Not reported | Not reported | Not reported | Not reported | 67.7  31.5  0.8 | 75.1  24.4  0.5 |
| **eGFR <60 ml/min/1.73 m^2^ (%)** | 25.9 | 20.1 | 7.4 | 21.8 | 58.9 | 89.1 | 40.6 | 48 |
| **HbA1c (%)** | 8.1 | 8.2 | 8.3 | 8.2 | 8.3 | Not reported | Not reported | Not reported |
| **Statin use (%)** | 77 | 75 | 75 | 82 | 69 | 64.8 | Not reported | Not reported |
| **MACE ^$^** | 0.86  (0.74–0.99) | 0.86  (0.75–0.97) | 0.93  (0.84–1.03) | 0.97  (0.85–1.11) | 0.80  (0.67–0.95) | N/A | Not reported | Not reported |
| **CV death** | 0.62  (0.49–0.77) | 0.87  (0.72–1.06) | 0.98  (0.82–1.17) | 0.92  (0.77–1.11) | 0.78  (0.61–1.00) | 0.81  (0.58–1.12) | 0.82  (0.69–0.98) | 0.92  (0.75 to 1.12) |
| **All-cause mortality** | 0.68  (0.57–0.82) | 0.87  (0.74–1.01) | 0.93  (0.82–1.04) | 0.93  (0.80–1.08) | 0.83  (0.68–1.02) | 0.69  (0.53–0.88) | 0.83  (0.71–0.97) | 0.92  (0.77 to 1.10) |
| **HF hospitalization** | 0.65  (0.50–0.85) | 0.67  (0.52–0.87) | 0.73  (0.61–0.88) | 0.70  (0.54–0.90) | 0.61  (0.47–0.80) | Not reported | 0.70  (0.59–0.83) | 0.69  (0.59 to 0.81) |
| **Hospitalization for HF or CV death^#^** | 0.66  (0.55–0.79) | 0.78  (0.67–0.91) | 0.83  (0.73–0.95) | 0.88  (0.75–1.03) | 0.69  (0.57–0.83) | 0.71  (0.55–0.92) | 0.75  (0.65–0.85) | 0.75  (0.65 to 0.86) |
| **Renal composite endpoint*** | 0.54  (0.40–0.75) | 0.60  (0.47–0.77) | 0.53  (0.43–0.66) | 0.81  (0.63–1.04) | 0.70  (0.59–0.82) | 0.61  (0.51–0.72) | 0.71  (0.44–1.16) | 0.50  (0.32 to 0.77) |

EMPA-REG OUTCOME, Empagliflozin cardiovascular outcome event trial in type 2 diabetes patients; CANVAS, canagliflozin cardiovascular assessment study; DECLARE-TIMI 58, Multicenter trial to evaluate the effect of dapagliflozin on the incidence of cardiovascular events; DAPA-HF, Study to evaluate the effect of dapagliflozin on the incidence of worsening heart failure; CREDENCE, Evaluation of the effects of canagliflozin on renal and cardiovascular outcomes in participants with diabetic nephropathy; VERTIS-CV, Cardiovascular outcomes following ertugliflozin treatment in type 2 diabetes mellitus participants with vascular disease.

ASCVD, atherosclerotic cardiovascular disease; eGFR, estimated glomerular filtration rate; MACE, major adverse cardiac event; HHF, hospitalization for heart failure; CV, cardiovascular; N/A, not available.

* renal endpoint reported mostly as a composite of sustained doubling of serum creatinine or a 40% decline in eGFR, end-stage kidney disease, or death of renal cause. The DAPA-HF renal composite endpoint was defined as a sustained decline in eGFR of 50% or greater, end-stage kidney disease, renal transplantation, renal death, and death from any cause. # Hospitalization for HF or CV death was a dual primary endpoint of the DECLARE-TIMI 58 trial. In EMPA-REG OUTCOME, this endpoint excluded fatal stroke events. The primary endpoint of DAPA-HF and EMPEROR-REDUCED was worsening heart failure (urgent heart failure visit or hospitalization for heart faliure) or cardiovascular death.

$ This outcome was the primary outcome for CANVAS and EMPA-REG OUTCOME and was a dual primary outcome for DECLARE-TIMI 58. It was a secondary outcome for CREDENCE. It consists of 3-point MACE, a composite of nonfatal myocardial infarct, nonfatal stroke, and cardiovascular death.
