# Supplementary material for: Sodium-glucose cotransporter 2 inhibitors: a practical guide for the Dutch cardiologist based on real-world experience
Source: Neth Heart J. 2021 Jun 16;29(10):490–9. doi: 10.1007/s12471-021-01580-9 (PMC8455761; doi:10.1007/s12471-021-01580-9)
Supplement: Supplementary file 2 — Table S2. Summary of currently available SGLT2 inhibitors in the Netherlands on indications, contra-indications, dosing, and most important side-effects [file 12471_2021_1580_MOESM2_ESM.docx]

**Table 2.** Doses, indications, contraindications and adverse effects of SGLT2 inhibitors

|  | **Empagliflozin**  **Jardiance®** | **Canagliflozin Invokana®** | **Dapagliflozin Forxiga®** | **Ertugliflozin Steglatro®** |
| --- | --- | --- | --- | --- |
| **Dose** | 10 mg once daily | 100 mg once daily | 10 mg once daily | 5 mg once daily |
| **Indications** | Improve glycemic control in adults with type 2 diabetes mellitus as an adjunct to diet, exercise, or other glucose-lowering drug  Reduce risk of myocardial infarct, stroke, or cardiovascular death in adults with type 2 diabetes mellitus and cardiovascular disease | | | |
|  | Reduce the risk of cardiovascular death and hospitalization for heart failure in adults with HFrEF | Reduce the risk of end-stage renal disease, doubling of serum creatinine, cardiovascular death, and hospitalization for HF in patients with type 2 diabetes mellitus and diabetic nephropathy with albuminuria (urine albumin/creatinine ratio >30) | Reduce the risk of cardiovascular death and hospitalization for heart failure in adults with HFrEF.  Reduce the risk of end-stage renal disease and doubling of serum creatinine in patients with chronic kidney disease |  |
| **Contraindications** | - Type 1 diabetes mellitus - History of serious hypersensitivity reaction to drug - Pregnancy or breastfeeding - eGFR <30 mL/min/1.73m^2^ - Malnutrion, dehydration and/or excessive alcohol abuse | | | |
| **Relative contraindications** | - Recurrent urogenital infections - Peripheral artery disease with foot ulcers | | | |
| **Adverse events** | - Genital fungal infections - Euglycemic diabetic ketoacidosis | | | |
